# Calcium Oxalate Differentiates Human Monocytes Into Inflammatory M1 Macrophages

**DOI:** 10.3389/fimmu.2018.01863

**Published:** 2018-08-22

**Authors:** Paul R. Dominguez-Gutierrez, Sergei Kusmartsev, Benjamin K. Canales, Saeed R. Khan

**Affiliations:** ^1^Department of Urology, University of Florida, Gainesville, FL, United States; ^2^Department of Pathology, Immunology and Laboratory Medicine, College of Medicine, University of Florida, Gainesville, FL, United States

**Keywords:** kidney stones, calcium oxalate, monocytes, macrophages, inflammatory cytokines, nephrolithiasis

## Abstract

**Purpose:**

A number of hyperoxaluric states have been associated with calcium oxalate (CaOx) deposits in the kidneys. In animal models of stone disease, these crystals interact with circulating monocytes that have migrated into the kidney as part of innate immunity. Similarly, macrophages surround CaOx crystals in kidneys of patients excreting high levels of oxalate. We investigate the effect of this exposure and subsequent human immunological response *in vitro*.

**Materials and methods:**

Primary human monocytes were collected from healthy donors and exposed to CaOx, potassium oxalate, and zinc oxalate (ZnOx). Cytokine production was measured with a multiplex ELISA. Quantitative reverse transcription-polymerase chain reaction was done to validate the mRNA profile expression. M1 macrophage phenotype was confirmed with immunofluorescence microscopy.

**Results:**

Both primary monocytes and THP-1 cells, a human monocytic cell line, respond strongly to CaOx crystals in a dose-dependent manner producing TNF-α, IL-1β, IL-8, and IL-10 transcripts. Exposure to CaOx followed by 1 h with LPS had an additive effect for cytokine production compared to LPS alone, however, LPS followed by CaOx led to significant decrease in cytokine production. Supernatants taken from monocytes were previously exposed to CaOx crystals enhance M2 macrophage crystal phagocytosis. CaOx, but not potassium or ZnOx, promotes monocyte differentiation into inflammatory M1-like macrophages.

**Conclusion:**

In our *in vitro* experiment, human monocytes were activated by CaOx and produced inflammatory cytokines. Monocytes recognized CaOx crystals through a specific mechanism that can enhance or decrease the innate immune response to LPS. CaOx promoted M1 macrophage development. These results suggest that monocytes have an important role promoting CaOx-induced inflammation.

## Introduction

A variety of crystals can form and accumulate within the kidney. These deposits can eventually lead to nephrolithiasis, a painful condition that is increasingly prevalent and costly around the world (1, 2). Calcium oxalate (CaOx) crystals are the major constituent of the most common kidney stones which start as deposits or plugs within the inner medullary collecting ducts or on subepithelial deposits of calcium phosphate on the renal papillae of patients with, among other abnormalities, high urinary oxalate levels or hyperoxaluria (3–7), which is most common in patients with primary hyperoxaluria. In rodents, hyperoxaluria leads to rapid crystallization of CaOx within the renal proximal tubular lumen (8, 9). Although most CaOx crystals move with the filtrate through the nephron, some crystals remain attached to tubular epithelial cells and appear to migrate into the renal interstitium. Over time, these crystals become surrounded, engulfed, and eventually cleared by tissue macrophages with minimal visible inflammatory changes. Similarly, Taguchi et al. recently demonstrated by renal papillary tip tissue biopsy that human CaOx stone formers have high amounts of tissue inflammatory markers but minimal visible inflammatory changes, suggesting that low-grade renal immune responses do occur in CaOx stone formers (10).

Tissue macrophages play important and complex roles in homeostasis, regulating immunity, inflammation, and angiogenesis while scavenging apoptotic cells (11). We have demonstrated *in vitro* that human macrophages are capable of engulfing, phagocytizing, and gradually disintegrating CaOx crystals and human stones fragments (12). These activated macrophages release an array of cytokines and chemokines intended to attract circulating monocytes to the site of tissue inflammation. Monocytes and macrophages have been implicated in CaOx stone disease in both humans and rodent models (13–16). Hyperoxaluric C57BL/6J mice expressed M-CSF and CCL2 which could potentially recruit monocytes (14); hyperoxaluric C57BL/6J mice that received M1 macrophage transfusions displayed increased CaOx production of IL-6 and TNFα compared to those that received M2 macrophage transfusions (15). In addition, M-CSF-deficient mice had significantly higher CaOx deposition in the kidneys than those of the wild-type mice (16). Furthermore, Okada et al. reported an overall increase in CD68 (+) macrophages in 60 patients who underwent radical nephrectomy for renal cancer who were then retrospectively classified as stone formers (13). Furthermore, Williams et al. demonstrated that monocytes isolated from the peripheral blood of CaOx stone formers (*n* = 12) displayed significantly lower mitochondrial maximal respiration, reserve capacity, and bioenergetic health index compared to healthy donors (17). To further our understanding of these immune responses, we exposed primary human monocytes and a monocyte cell line (THP-1 cells) to soluble oxalate, CaOx crystals, and a variety of other minerals and controls to determine response, time-dependent effects, and inflammatory cytokine and chemokine production.

## Materials and Methods

### Reagents and Culture Media

Ultrapure 99.999% CaOx (Alfa Aesar), potassium oxalate (K_2_Ox), zinc oxalate (ZnOx), and 200 nm or smaller hydroxyapatite (HA) crystals were purchased from Sigma-Aldrich. 500–1,000 ng/ml LPS from *S. enterica* serotype Minnesota Re595 (LPS Se, TLR4 ligand, Ultrapure grade, Sigma-Aldrich) was used as a positive control for innate immune stimulation (18–21). *In vitro* experiments were conducted using RPMI 1640 medium supplemented with 10% fetal bovine serum, 20 mM HEPES, 20 mM sodium pyruvate, and 100 U/ml penicillin–streptomycin (Hyclone Laboratories, Logan, Utah).

### Cell Culture

Human monocytic cell line THP-1 cells were obtained from the American Type Culture Collection (ATCC, Manassas, VA, USA). For analysis of THP-1 monocyte response to crystals *in vitro*, log phase cells were seeded at 1 × 10^6^ cells/ml in a 24-well plate.

### Preparation of Primary Human Monocytes

Following institutional review board approval, human buffy coat samples without demographic data were obtained from LifeSouth Community Blood Center (Gainesville, FL, USA). Peripheral blood mononuclear cells (PBMCs) were separated by Lymphoprep (Accu-Prep, 1.077 g/ml, Oslo, Norway) gradient density centrifugation per manufacturer’s recommendations. PBMCs were washed twice with 10 ml of PBS, and red blood cells were lysed using ACK lysing buffer (BioWhittaker, Walkersville, MD, USA). Monocytes were purified from PBMC using the MACS method (Miltenyi Biotec, Bergisch Gladbach, Germany) per manufacturer’s instructions. Briefly, cells were incubated with beads conjugated with anti-CD14 and positively selected using LS columns (Miltenyi Biotec). 95% of recovered cells expressed monocyte marker CD14.

### Preparation of Primary Human Macrophages

#### Cytokine-Induced Differentiation

Monocytes were differentiated into macrophages over 6 days by seeding in 24-well culture plate (1 × 10^6^ cell/ml) in complete RPMI 1640 culture medium and treating with 20 ng/ml of either recombinant human M-CSF or recombinant human GM-CSF at day 0 and day 3.

#### CaOx-Induced Differentiation

Monocytes were seeded in 24-well culture plate (1 × 10^6^ cell/ml) in complete RPMI 1640 culture medium and exposed to 0.05 mM (64.0 μg/ml) or 2.5 mM (320 μg/ml) CaOx.

### Phagocytosis Assay Using Qdot525 Labeled CaOx

Primary human monocytes were stimulated with 2.5 mM CaOx, HA, or PBS as a control for 18 h. Supernatants were collected and centrifuged to pellet any mineral, cells, and debris. Clean supernatant was frozen at −80°C. Autologous monocytes were differentiated into M2 macrophages as described above. At day 6, media was removed and a solution of half fresh media and half supernatants from the monocyte treatments was added. After 8 h, 1.56 mM (200 mg/ml) Qdot 525-labeled CaOx was added. After 1 h, macrophages were washed three times with PBS to remove any extracellular CaOx. CaOx was labeled with Qdot 525 amino quantum dots (Qdot525, Applied Biosystems) following a modified manufacturer’s protocol. 100 μl of Qdot525 was mixed with 1.0 ml of borate buffer and 0.5 ml of 2.50 mM (320 μg/ml) CaOx in 10 nM borate buffer. At 4^o^C, 10 μl of 10 mg/ml 1-Ethyl-3-(3-dimethylaminopropyl) carbodiimide (Thermo Scientific, Waltham, MA, USA) was added to the above and incubated for 1 min. Following incubation, Qdot525-CaOx was washed three times with PBS, pelleted, and resuspended in 1 ml of fresh media. At 20× magnification, EVOS FL Cell Imaging System (Applied Biosystems) evaluated intercellular uptake of CaOx crystals. Macrophages positive for Qdot 525-labled CaOx were visually determined using photoshop. *N* = 3 for each treatment group.

### Immunofluorescence and HEMA 3 Staining of Macrophages

Macrophages were fixed with 4% paraformaldehyde and 1% glutaraldehyde for 10 min. Followed by rinsing and blocking with 2% BSA, macrophages were stained with primary antibodies for M1 markers CD68 (Abcam ab955) and CD86 (Abcam, ab53004) and M2 markers CD163 (Abcam, ab87099), CD206 (Abcam, ab8918), and phosphorylated STAT6 (Abcam, ab28829). Secondary antibody staining was done with Alexa Fluor 488, goat anti-mouse IgG (Abcam, ab150113) and Cy5, and goat anti-rabbit IgG (Abcam, ab6563). PROTOCOL Hema 3 staining systems (Fisher Scientific) were used to stain macrophage morphology according to the manufacturer’s protocol.

### Quantitative Real-Time PCR (qPCR)

Total cellular RNA was isolated from monocytes using Direct-zol RNA MiniPrep (Zymo Research) according to the manufacturer’s instructions. RNA concentration was measured with a Take3 plate on a Synergy H1 plate reader (BioTek). cDNA for each RNA sample was synthesized in 20 µl reactions using the High-Capacity cDNA Reverse Transcription Kit (Applied Biosystems) following the manufacturer’s protocol. qPCR analysis was performed using a 7900HT Fast Real-Time PCR System (Applied Biosystems). cDNA-specific TaqMan Gene expression assays for human TNFα (assay ID: Hs01113624_g1), IL-1β (assay ID: Hs01555410_m1), IL-6 (assay ID: Hs00985639_m1), IL-8 (assay ID: Hs00174103_m1), and CCL2 (assay ID: Hs00234140_m1) from Applied Biosystems were used in the study. A Eukaryote 18s rRNA (assay ID: 4319413E) was used as an endogenous control. Relative expression of mRNA was determined by the ΔΔC_T_ method where the cycle threshold (C_T_) values were determined for the mRNA expression relative to untreated controls.

### Multiplex Cytokine ELISA Assay

Cell-free cell culture supernatant samples were stored at −20°C until analyzed. The system used was a multiplex ELISA assay manufactured by Meso Scale Discovery (MSD, Gaithersburg, MD, USA) containing 10 individual ELISAs per well. Supernatants were tested with a U-PLEX Proinflammatory Panel1 Human Kit [a multiplex 96-well ELISA plate based assay that contained primary antibodies to IL-12, IL-10, TNFα, IL-6, IL22, interleukin 1 receptor antagonist (IL-1Ra), interferon alpha 2a (IFNα2a), interferon beta (IFNβ), interferon gamma (IFNγ), and IL-1β] per manufacturer’s recommendations. Briefly, the MSD plex assays were run as follows. Calibration curves were prepared in the supplied assay diluent for human serum, with a range of 40,000–1.2 pg/ml, depending on the cytokine. Arrays were preincubated with 25 µl per well of assay diluent for 30 min. After the pre-incubation, 25 μl sample or calibrator was added in duplicate to the appropriate wells. The array was then incubated at room temperature for 2 h with primary antibody. The array was washed with PBS plus 0.05% Tween 20, and 25 µl detection antibody reagent was added. After 2 h of incubation at room temperature with secondary antibody, the array was washed, and the detection buffer was added. Results were read with a QuickPlex SQ 120. Sample cytokine concentrations were determined using MSD Discovery Workbench 4.0 software.

### Data Analysis

Analyses were performed using JMP Pro version 10 (SAS, Cary, NC, USA). Dunnett’s with Control and Tukey-Kramer Honest Significant Difference test was used to evaluate significance. *P* value less than 0.05 was considered significant.

## Results

### CaOx Stimulates the Production of Inflammatory Cytokines in THP-1 Cells

**Figure 1:**
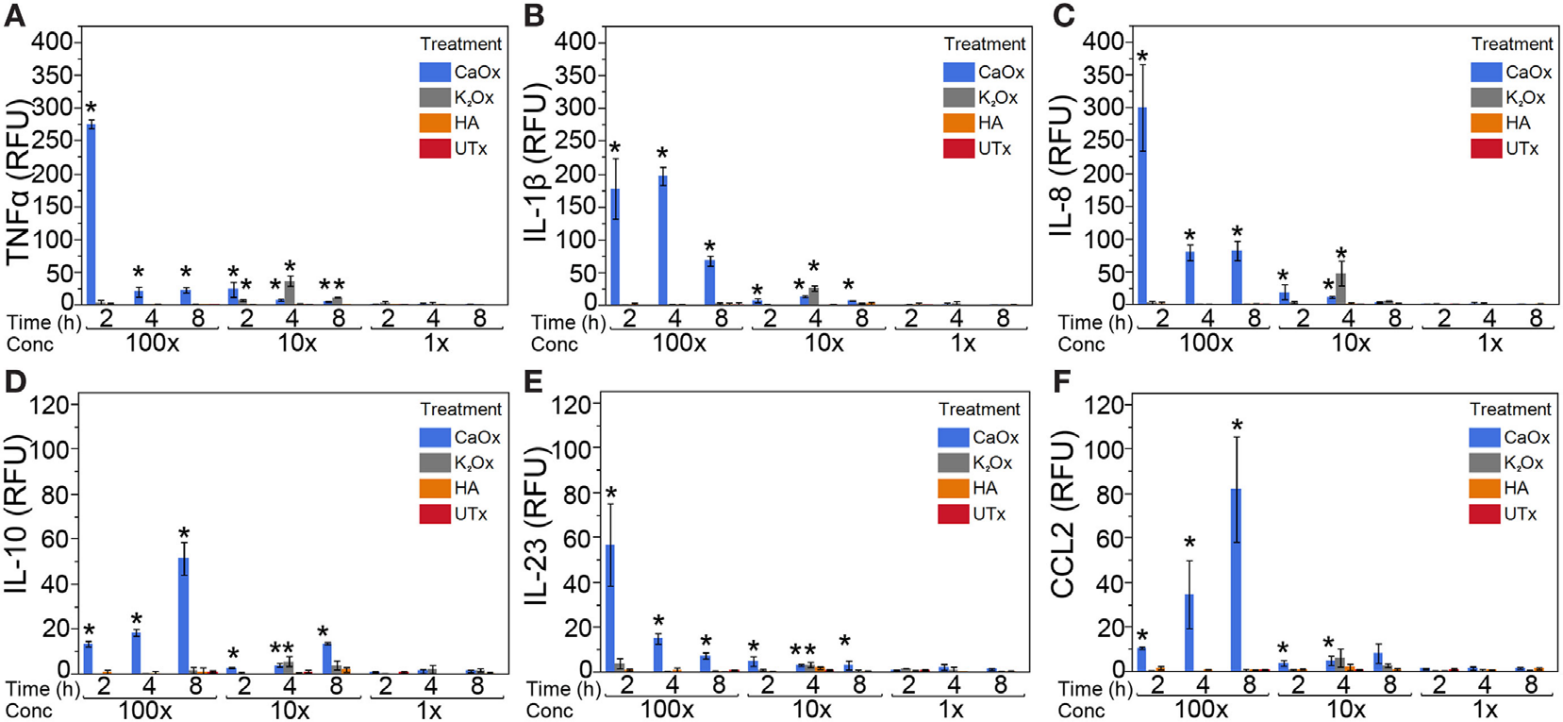
Calcium oxalate (CaOx) induces a dose- and time-dependent expression of inflammatory cytokines in the human monocytic THP-1 cell line. THP-1 cells were treated with molar 100× (7.81 mM, 1,000 µg/ml), 10× (0.78 mM, 100.0 µg/ml), and 1× (0.078 mM, 10.0 µg/ml) CaOx (blue); 100× (6.84 mM, 1,138 µg/ml), 10× (0.68 mM, 113.8 µg/ml), and 1× (0.068 mM, 11.4 µg/ml) potassium oxalate (gray, K_2_Ox); 100× (1.99 mM, 1,000 µg/ml), 10× (0.20 mM, 100.0 µg/ml), and 1× (0.020 mM, 10.0 µg/ml l) hydroxyapatite (orange, HA); or untreated controls (red, UTx). Following treatment, cells were incubated for 2, 4, or 8 h. Total RNA was purified from respective cell pellets and analyzed by quantitative real-time PCR for relative fold expression of TNFα **(A)**, IL-1β **(B)**, IL-8 **(C)**, IL-10 **(D)**, IL-23 **(E)**, and CCL2 **(F)**. mRNA expression was normalized with 18s RNA respectively. All results are expressed as relative fold change units (RFU) ± SD from four independent experiments. *p < 0.05 compared to UTx.

Human monocytic THP-1 cells were exposed to 100, 10, and 1× dose taper of CaOx (Figure 1, blue bars), K_2_Ox (Figure 1, gray bars), or HA (Figure 1, orange bars) over a period of 8 h. When exposed to CaOx crystals, THP-1 cells upregulated the expression (*p* < 0.05) of TNFα, IL-1β, IL-8, IL-10, IL-23, and CCL2 (monocyte chemotactic protein-1/MCP-1) in a time and dose-dependent manner (Figure 1, blue bars). However, THP-1 cells did not display a dose-dependent response to soluble K_2_Ox and showed a rather moderate time-dependent response (Figure 1, gray bars). HA failed to stimulate a significant immune response (Figure 1, orange bars).

### Primary Human Monocytes Respond to CaOx but Not HA

**Figure 2:**
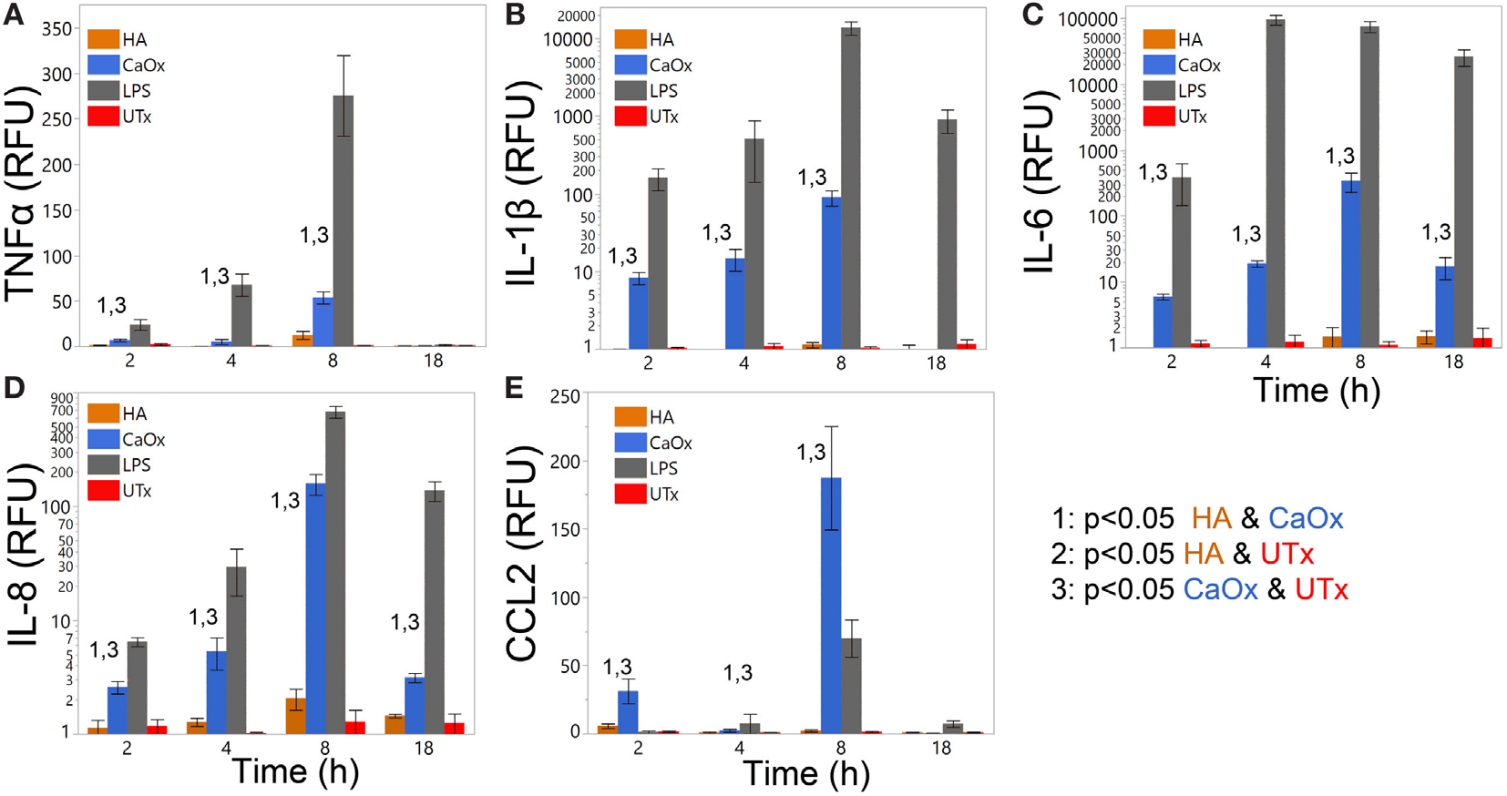
Calcium oxalate (CaOx) but not hydroxyapatite (HA) stimulates proinflammatory cytokine expression in primary human monocytes in a time-dependent manner. Primary human monocytes cells were treated with 1.95 mM (250 µg/ml) CaOx (blue), 0.40 mM (200 µg/ml) HA (orange), 1 µg/ml LPS (gray, positive control), and UTx (red, untreated) and incubated for 2, 4, 8, or 18 h. Total RNA was purified from respective cell pellets and analyzed by quantitative real-time PCR for relative fold expression of TNFα **(A)**, IL-1β **(B)**, IL-6 **(C)**, IL-8 **(D)**, and CCL2 **(E)**. mRNA expression was normalized with 18s RNA respectively. All results are expressed as relative fold change units (RFU) ± SD from four independent experiments. *p < 0.05 compared to UTx.

Primary human monocytes were treated with CaOx (Figure 2, blue bars) and assayed for expression changes. TNFα, IL-1β, IL-6, IL-8, and CCL2 increased significantly (*p* < 0.05) in monocytes treated with CaOx; however, HA (Figure 2, orange bars) had no significant effect. LPS (gray bars) and PBS (UTx, red bars) were used as positive and negative controls, respectively (Figure 2).

### CaOx Alters Monocytes’ Response to LPS Exposure

**Figure 3:**
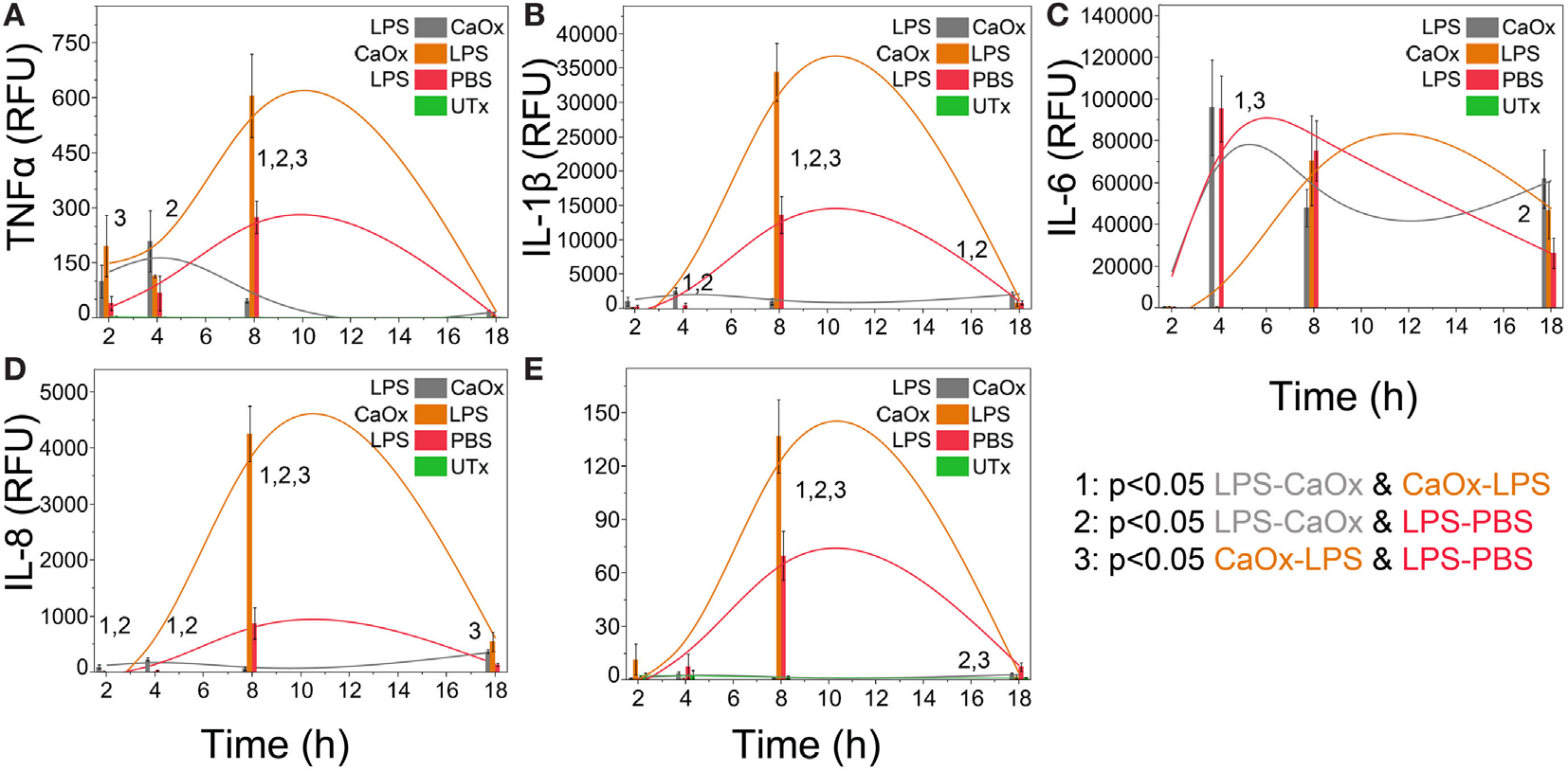
Calcium oxalate (CaOx) upregulates or downregulates inflammatory cytokine expression in monocytes response to LPS depending on order of exposure. Primary human monocytes cells were incubated for 1 h with 500 ng/ml LPS, 1.95 mM (250 µg/ml) CaOx, or PBS. After incubation, monocytes were exposed to 1.95 mM CaOx (gray), 500 ng/ml LPS (orange), or 500 ng/ml LPS (red), respectively and incubated for 2, 4, 8, or 18 h. Untreated samples (UTx, green) only received PBS for both incubations. Total RNA was purified from respective cell pellets and analyzed by quantitative real-time PCR for relative fold expression of TNFα **(A)**, IL-1β **(B)**, IL-6 **(C)**, IL-8 **(D)**, and CCL2 **(E)**. mRNA expression was normalized with 18s RNA respectively. All results are expressed as relative fold change units (RFU) ± SD from four independent experiments. Tukey-Kramer Honest significant difference test was used to determine significance for multiple comparisons.

Primary human monocytes exposed to LPS followed 1 h later by CaOx (Figure 3, gray bars) displayed a significant decrease (*p* < 0.05) of TNFα, IL-1β, IL-8, and CCL2 (Figures 3A,B,D,E) compared to LPS alone (Figure 3, red bars) and CaOx followed by LPS exposure (Figure 3, orange bars). Also, CaOx followed by LPS (orange) exposure displayed significantly higher levels of expression for these cytokines and chemokines (*p* < 0.05) than LPS alone (Figures 3A,B,D,E red bars). IL-6 appears to be primarily driven by LPS; however, CaOx followed by LPS exposure appears to have a delayed IL-6 response compared to the other LPS exposures (Figure 3C).

### Monocytes Exposed to CaOx Enhanced M2 Macrophage Uptake of CaOx Crystals

**Figure 4:**
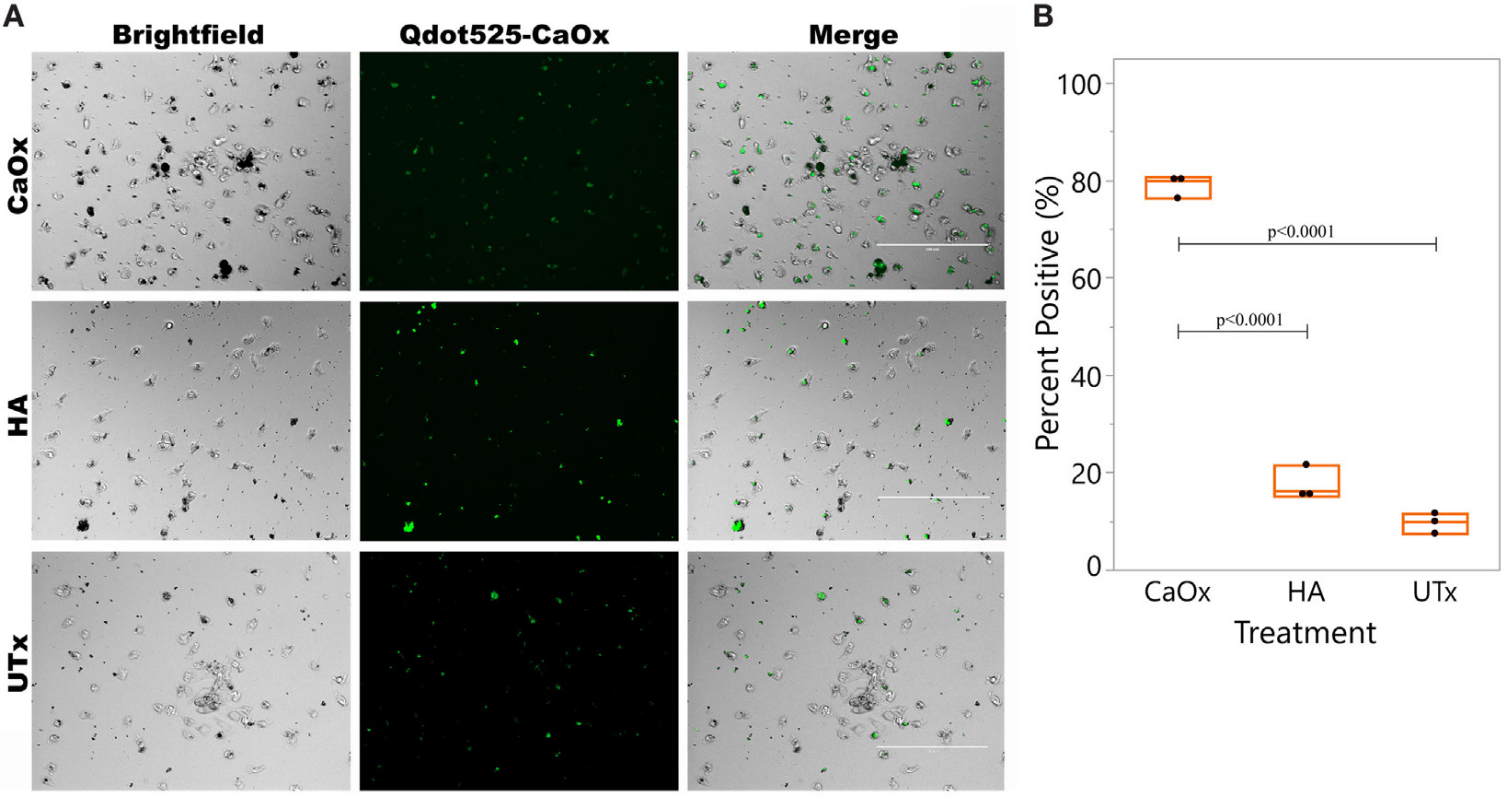
CaOx stimulated monocytes to enhance M2 macrophage uptake of CaOx. **(A)** Primary monocytes were treated with M-CSF for 6 days to differentiate into M2 macrophages. Donor matched monocytes were exposed to 2.50 mM calcium oxalate (CaOx), hydroxyapatite (HA), or untreated (UTx, PBS) and incubated for 24 h. Supernatants from monocytes was transferred to the M2 macrophages and incubated for 2 h. After 2 h, macrophages were exposed to 1.56 mM Qdot525 labeled CaOx and uptake was imaged after 1 h with an Evos FL Auto microscope GFP light cube. **(B)** 79.0 ± 2.30% of macrophages exposed to CaOx supernatant had phagocytosed Qdot525 labeled CaOx compared to 17.6 ± 3.50% of macrophages exposed to HA supernatant (*p* < 0.0001) and 9.70 ± 2.31% of UTx (*p* < 0.0001). Images are representative of *n* = 3 at 20× magnification.

Primary human monocytes were exposed to CaOx, HA, or untreated (UTx) for 24 h. Matched donor primary monocytes were differentiated into M2 macrophages with M-CSF. The culture supernatants were transferred to matched donors of primary human M2 macrophages 2 h prior to CaOx exposure (Figure 4). Within 1 h of exposure, macrophages (79.0 ± 2.30%) pretreated with supernatant from CaOx-treated monocytes displayed significantly greater uptake than macrophages pretreated with supernatant from HA-treated (17.6 ± 3.50%, *p* < 0.0001) or UTx (9.70 ± 2.31%, *p* < 0.0001) monocytes (Figure 4).

### Macrophage Differentiation Is Specific to CaOx and Not to K_2_Ox or ZnOx

**Figure 5:**
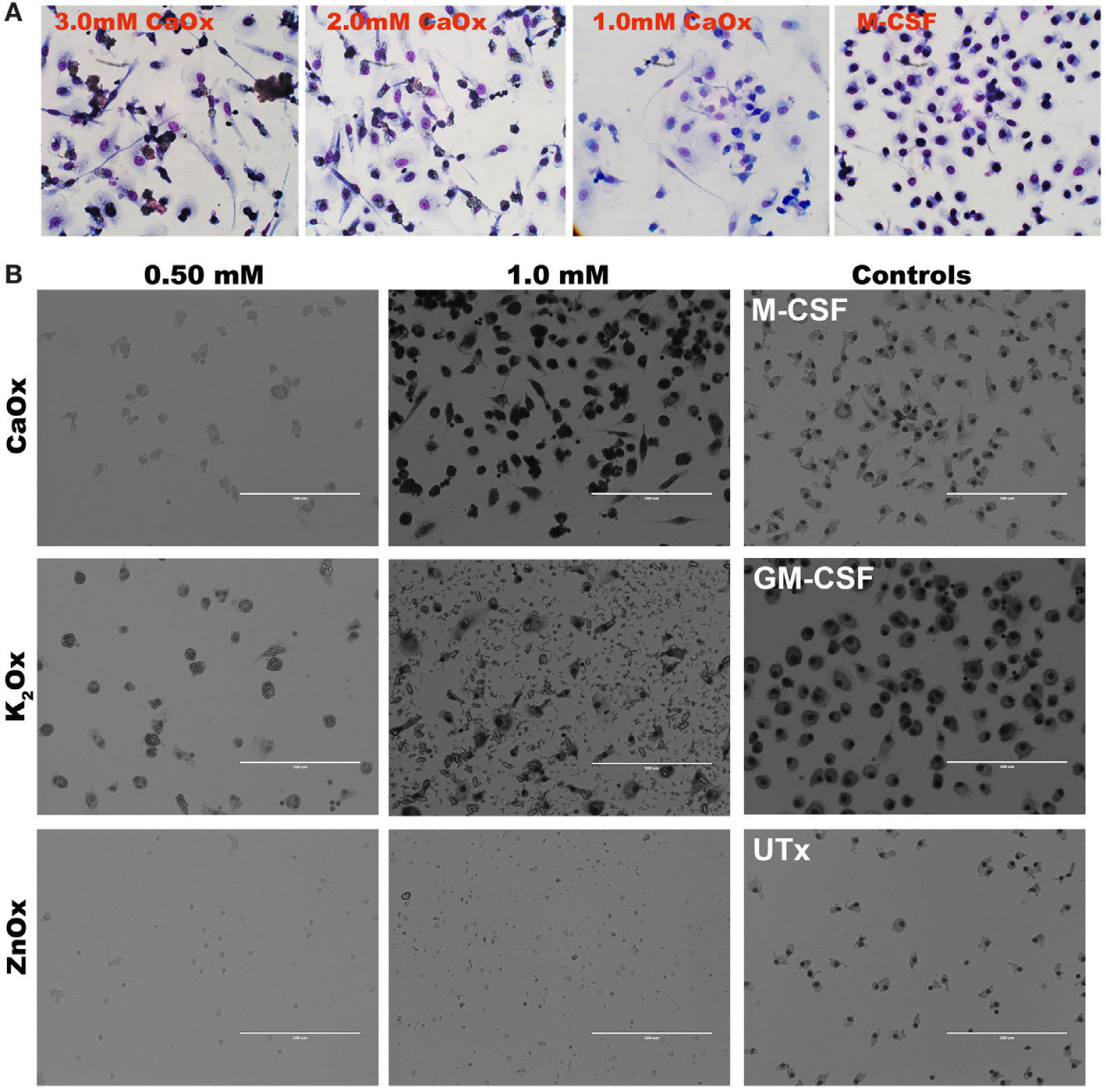
Calcium oxalate (CaOx) induces macrophage-like morphology in 3 days. **(A)** Primary monocytes were exposed to 1.0, 2.0, or 3.0 mM CaOx, or 20 ng/ml M-CSF for 3 days. After day 3, cells were stained with HEMA 3 kit for macrophage morphology. **(B)**. Primary human monocytes were exposed to 0.50 or 1.00 mM of CaOx, potassium oxalate (K_2_Ox), or zinc oxalate (ZnOx) and incubated for 6 days. Images are representative of *n* = 3 at 20× magnification.

Primary human monocytes were exposed to CaOx over a period of 6 days. At day 3 of CaOx exposure, monocytes stained with HEMA 3 displayed macrophage-like morphology similar to GM-CSF-treated monocytes (Figure 5A). To validate CaOx specificity, primary monocytes were exposed to CaOx, K_2_Ox, or ZnOx over a period of 6 days (Figure 5B). ZnOx did not induce differentiation of primary monocytes; however, some monocytes displayed macrophage-like morphology when exposed to K_2_Ox (Figure 5B). K_2_Ox and UTx displayed a minimal number of macrophages. M-CSF- and GM-CSF-induced macrophages were positive controls and formed M2 and M1 macrophages, respectively.

### CaOx Induces Inflammatory M1-Like Macrophage Differentiation and Cytokine/Chemokine Production

**Figure 6:**
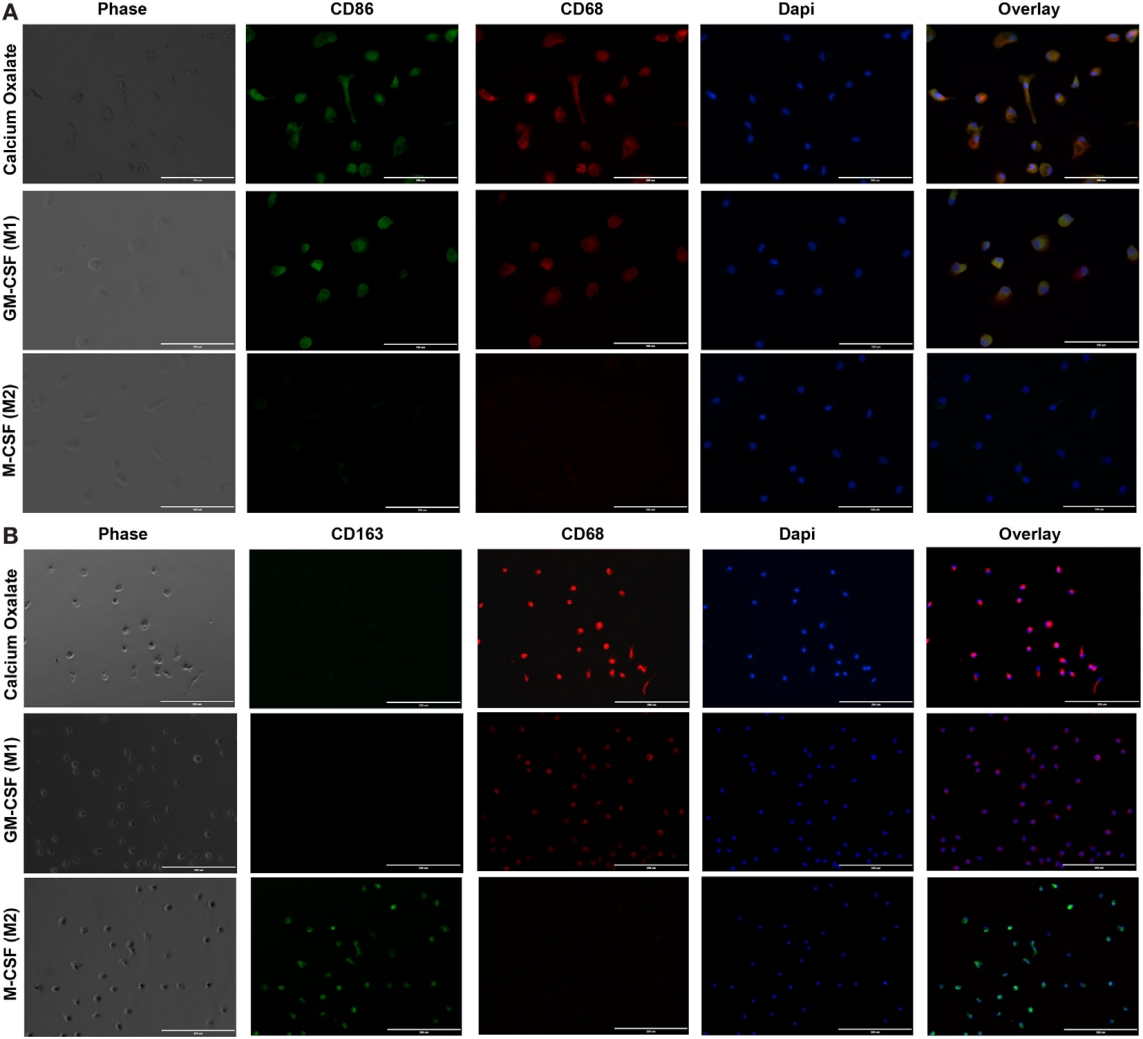
Calcium oxalate (CaOx) induces monocytes to differentiate into inflammatory (M1) macrophages. Primary monocytes were exposed to 1.95 mM CaOx, 20 ng/ml M-CSF (M2 control), or GM-CSF (M1 control) and incubated for 6 days. **(A)** After day 6, cells were fixed and stained with rabbit anti-CD86 (green) and mouse anti-CD68 (red) (40×, bar, 100 µm) or **(B)** double stained with rabbit anti-CD163 (green) and mouse anti-CD68 (red) (20×, bar, 200 µm). Nuclei were counterstained by 4,6-diamidino-2-phenylindole (DAPI, blue). N = 3.

Primary human monocytes were again exposed to CaOx over a period of 6 days. M-CSF- and GM-CSF-induced macrophages were positive controls and form M2 and M1 macrophages respectively. CaOx differentiated macrophages and GM-CSF-induced (M1) macrophages were CD86 and CD68 double positive compared to M-CSF induced (M2) macrophages that were double negative (Figure 6A). Macrophages were also double stained for CD163 and CD68 or CD206 (mannose receptor) and phosphorylated (p)STAT6. Both CaOx induced and GM-CSF-induced macrophages were negative for CD163, CD206, and pSTAT6; however, M-CSF induced macrophages where positive for all three markers (Figure 6B; Figure S1 in Supplementary Material).

### CaOx-Induced Macrophages Produce M1-Like Macrophage Cytokine and Chemokine Profiles

**Figure 7:**
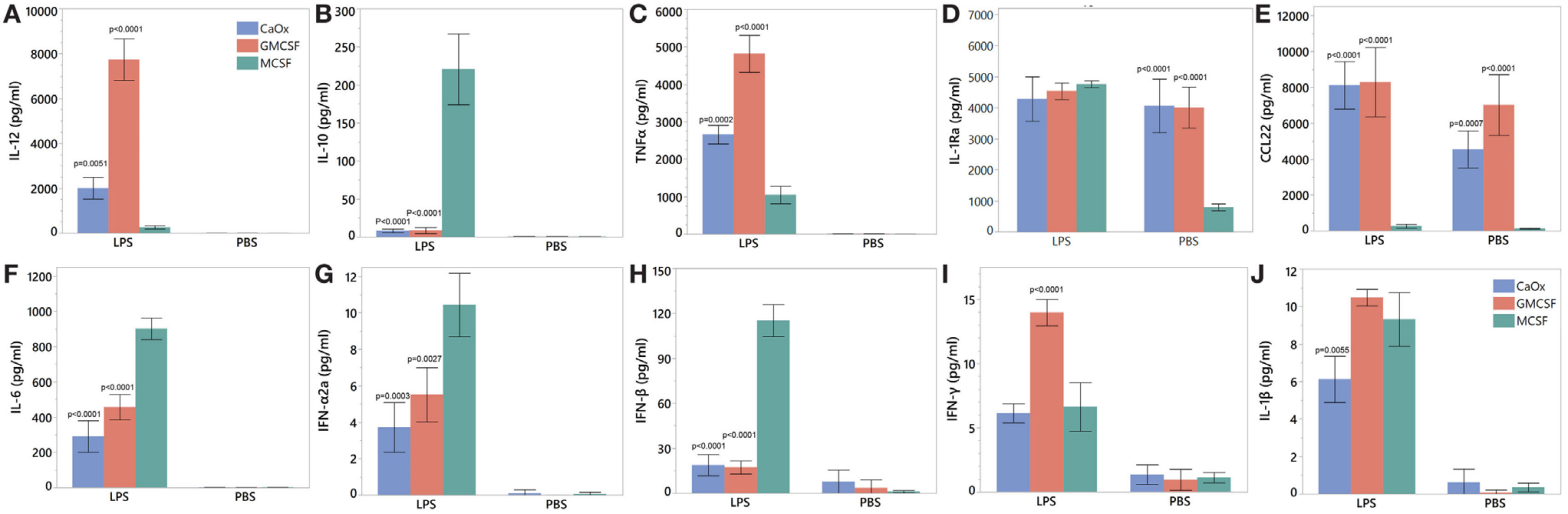
Cytokine profile of calcium oxalate (CaOx)-induced inflammatory (M1) macrophages after stimulation with LPS. Primary monocytes were exposed to 1.95 mM CaOx, 20 ng/ml M-CSF (M2 control), or GM-CSF (M1 control) and incubated for 6 days. At day 6, macrophages were exposed to 1 μg/ml LPS or PBS (control) and incubated for 24 h. Supernatant was collected at 24 h and for IL-12 **(A)**, IL-10 **(B)**, TNFα **(C)**, IL-6 **(D)**, IL22 **(E)**, interleukin 1 receptor antagonist **(F)**, interferon alpha 2a **(G)**, interferon beta **(H)**, interferon gamma **(I)**, and IL-1β **(J)** protein analysis using Meso Scale Discovery U-Plex (10 multiplex) ELISA. All results are expressed as pg/ml ± SD from four independent experiments. *p < 0.05 compared to M-CSF.

M1 marker IL-12 was significantly higher for both CaOx (*p* = 0.0051) and GM-CSF (*p* < 0.0001) induced macrophages compared to M-CSF induction after LPS treatment; M2 marker IL-10 was significantly lower for both CaOx (*p* < 0.0001) and GM-CSF (*p* < 0.0001) induction compared to M-CSF induction (Figures 7A,B). After LPS exposure, TNFα (*p* = 0.0002, *p* < 0.0001) and CCL22 (*p* < 0.0001, *p* < 0.0001) were significantly higher in CaOx and GM-CSF-induced macrophages, but significantly lower for IL-6 (*p* < 0.0001, *p* < 0.0001), IFNα2a (*p* = 0.0003, *p* = 0.0027), and IFNβ (*p* < 0.0001, *p* < 0.0001) compared to M-CSF induced macrophages (Figures 7C,E–H). For the PBS control, CCL22 (*p* = 0.0007, *p* < 0.0001) and IL-1Ra (*p* < 0.0001, *p* < 0.0001) were significantly higher in CaOx and GM-CSF-induced macrophages (Figures 7D,E). INFγ (*p* < 0.0001) was significantly higher in LPS-stimulated GM-CSF-induced macrophages (Figure 7I). IL-1β (*p* = 0.0055) significantly lower in LPS-stimulated CaOx-induced macrophages (Figure 7J).

## Discussion

Since this study attempts to model crystal and immune cell interactions *in vitro*, a critical appraisal of our methodology is important. First, in order to standardize the exposure of oxalate between the different mineral types, molarity was used. Because CaOx (CaC_2_O_4_) and K_2_Ox (K_2_C_2_O_4_) differ in molecular weight, 1,000 μg/ml of CaOx contains a greater number of oxalate molecules than 1,000 μg/ml of K_2_Ox. Therefore, to achieve 7.81 mM oxalate using CaOx and K_2_Ox, more K_2_Ox (1,438 µg/ml) is needed than CaOx (1,000 µg/ml). Second, to control for calcium molecules, molarity (mols of calcium per unit volume) was used. For example, 1 mM HA (HCa_5_O_13_P_3_) has five times the number of calcium atoms than 1 mM CaOx (CaC_2_O_4_). Thus, the variations in concentration between experiment and controls are an attempt to control for mineral concentration, so mM and mg/ml values are used when possible.

The biological levels of oxalate vary according to the biological fluid. Serum oxalate in normal individuals range from 0.2 to 10 µmol/l and in renal failure is as high as 89 µmol/liter (5, 22–24). For children with primary hyperoxaluria type 1, serum levels have been reported to be 125.7 µmol/l (25). In the saliva of 41 healthy males and 40 females, oxalate was reported to range from 0.10 ± 0.09 and 0.18 ± 0.17 µM, respectively (26). Oxalate in tartar was reported to be 3.3 ± 1.2 mmol/kg tartar (26). In urine, normal individuals excrete 0.222–0.444 mmol (20–40 mg) of oxalate daily; however, for individuals with primary hyperoxaluria, urinary oxalate excretion is 1.5–3.0 mmols (135–270 mg) per day (27). To establish the optimal response of monocytes to oxalate, we tested a range from as high as 7.81 mM (1,000 µg/ml) to 0.50 mM (64.0 µg/ml) CaOx.

In our *in vitro* model, CaOx crystals stimulate a significant, finely regulated, dose- and time-dependent release of TNFα, IL-1β, IL-6, IL-8, and CCL2 (Figures 1 and 2) from both human monocytes and THP-1 cells, similar to the innate immune response seen after LPS or peptidoglycan exposure (18, 28, 29). Consistent with Williams et al. results (17), we also demonstrate IL-6 expression in primary human monocytes (Figure 2C, blue bars). THP-1 cells expressed IL-23 and IL-10; however, primary human monocytes did not express IL-23 and IL-10. THP-1 cells are a human monocytic cell line derived from a 1-year-old male with acute monocytic leukemia (30). Though they are similar to primary monocytes, their response can differ. Sintiprungrat et al. reported IFNα expression by U937 human monocytic cells when exposed to CaOx (31). However, THP-1 cells and primary human monocytes did not express IFNα, IFNβ, and IFNγ that could be detected by qPCR (data not shown). Exposing THP-1 cells to K_2_Ox did not cause a response (Figure 1, gray bars), but over time, K_2_Ox precipitated to form CaOx (Figure 5B), which was likely responsible for the small THP-1 response noted using 10× concentration (Figure 1, gray bars). HA caused no response in THP1 cells (Figures 1 and 2, orange bars). Similarly, neither K_2_Ox nor ZnOx drove macrophage differentiation compared to CaOx (Figure 5); it appears that human monocytes recognize CaOx crystals, but not oxalate or hydroxyapatite.

With the exception of IL-6, the cytokines TNFα and IL-1β, and the chemokines IL-8 and CCL2 have dramatically different responses depending on if the primary human monocytes first encountered CaOx or LPS. Monocytes exposed to CaOx followed by LPS (CaOx-LPS, Figure 3, orange bars) displayed a greater response than that of LPS alone (Figure 3, red bars), suggesting that oxidative stress caused by CaOx enhances toll-like receptor 4 activation in monocytes (32, 33). However, the reverse leads to an unpredicted response: monocytes exposed to LPS followed by CaOx (LPS-CaOx, Figure 3, gray bars) display decreased expression of cytokines and chemokines compared to CaOx followed by LPS (Figure 3, orange bars) or LPS alone (Figure 3, red bars). This type of response resembles LPS tolerance, where low doses of LPS exposure attenuate subsequent responses to higher doses of LPS (18, 28, 29). Li et al. reported that the calcium sensing receptor (CaSR) recognized CaOx in HK-2 cells and promoted crystal adhesion in rat kidneys (34). Kelly et al. reported that the CaSR inhibited LPS activation of NFκB and TNFα secretion in mouse peritoneal macrophages when they were pre-exposed to calcium (35). However, we observed the decreased effect in reverse where the monocytes were pre-exposed to the LPS followed by CaOx (LPS-CaOx). When we exposed primary human monocytes to HA followed by LPS, cytokine and chemokine expression were not affected (data not shown). This may be due to the CaSR only recognizing calcium ions. CaOx and HA are highly insoluble with solubility product constants (Ksp) of 2.7 × 10^−9^ and 1 × 10^−34^ respectively. CaOx may act as a type of “binary switch,” triggering two different intra-cellular pathways with the same signal. This phenomenon has been reported when monocytes were exposed to monosodium urate (MSU) crystals alone (inflammatory pathways through NALP3) or MSU crystals in the presence of dead cells (sterile inflammatory pathway *via* Clec12a) (36). This unique response may have an important role in the gut. The gut is under a constant burden of LPS and periodic burden of CaOx. Foods such as spinach contain 400–900 mg of oxalate; for example, 100 g of New Zealand spinach is reported to have 736 mg of soluble oxalate and 220 mg of CaOx (37–39). Most of the oxalate absorbed is excreted in the urine, but 10% or less is secreted back into the intestine (40). The LPS-CaOx response would simulate what happens when an individual consumes a food such as spinach where the gut is in constant exposure to LPS and other bacterial ligands and only periodically exposed to high amounts of oxalate. The LPS-CaOx response would be evolutionarily beneficial. The inflammatory response from CaOx or CaOx-LPS would be detrimental and potentially lead to inflammatory bowel disease. It is interesting to note that CaOx stone disease is highly associated with IBD (41–43).

In our experiment, monocytes respond to CaOx by producing inflammatory cytokines, such as tumor necrosis factor-alpha, IL-1β, and IL-6, and chemokines, such as CCL2. These signals activate and recruit circulating monocytes and tissue macrophages to promote CaOx clearance (12). Consistent with our previous work, the supernatant from monocytes previously exposed to CaOx crystals enhanced M2 macrophage phagocytosis of CaOx (Figure 4) (12). CaOx alone causes the monocytes to undergo differentiation into macrophages (Figures 5 and 6). Since neither K_2_Ox nor ZnOx was able to drive differentiation, monocytes likely utilize a receptor that preferentially recognizes the CaOx crystals.

Calcium oxalate and GM-CSF (positive control) macrophages were positive for M1 macrophage markers CD68 and CD86 and negative for M2 markers CD163, CD206, and pSTAT6 (Figure 6A; Figure S1 in Supplementary Material). CaOx and GM-CSF produce proinflammatory cytokines IL-12 (M1 signature marker) and TNFα when stimulated with LPS (Figures 7A,C). CCL22, regardless of LPS treatment, appears to be an M1 marker and is highly elevated by both GM-CSF and CaOx exposure (Figure 7E). The CaOx and GM-CSF-induced macrophages were negative for CD163 (M2 marker) unlike the M-CSF induced macrophages (Figure 6B). After LPS stimulation, IL-6, IFNα2a, and IFNβ were significantly higher in the M-CSF induced macrophages than in the CaOx and GM-CSF-induced macrophages (Figures 7F–H). IL-1Ra is an antagonist of IL-1α and IL-1β and protects the host from endotoxin-induced injury (44, 45). We have previously shown that IL-1Ra was secreted by M2 macrophages in response to CaOx and human CaOx kidney stones (12). CaOx, GM-CSF, and M-CSF macrophage phenotypes responded to LPS stimulation by producing IL-1Ra; however, IL-1ra was significantly elevated in the unstimulated CaOx and GM-CSF-induced macrophages compared to M-CSF-induced macrophages (Figure 7D). IFNγ appears to be specific to LPS-stimulated GM-CSF-induced macrophages (Figure 7I). Overall, these results indicate that CaOx-induced macrophages display an M1 macrophage phenotype.

## Conclusion

Human monocytes and human monocyte cell lines respond in a specific manner to the CaOx crystal, eliciting a local, tissue inflammatory response that drives M1 macrophage differentiation. This response was not seen after exposure to hydroxyapatite crystals or various oxalate controls. The unique and opposite response of monocytes to LPS-CaOx and CaOx-LPS appears to indicate the existence of CaOx receptor with a binary switch function, similar to what has been described for MSU receptors. Prolong exposure to CaOx induces monocytes to differentiate into M1 macrophages. Further investigation into the signaling mechanism is needed.

## Human and Animal Rights

Following institutional review board approval, human buffy coat samples were purchased from LifeSouth Community Blood Centers. Buffy coats were received de-identified without any donor information. No animals were used for this study.

## Consent Form

Following institutional review board approval, this study did not require a consent form, since no human donors were recruited for this study.

## Author Contributions

PD-G: project development, data collection, data analysis, manuscript writing, and manuscript editing. SK: project development and manuscript editing. BC: project development and manuscript editing. SRK: project development and manuscript editing.

## Conflict of Interest Statement

The authors declare that the research was conducted in the absence of any commercial or financial relationships that could be construed as a potential conflict of interest.

## Supplementary Material

Figure S1 Calcium oxalate (CaOx) induced macrophages lack M2 macrophage markers phosphorylated STAT6 and CD206. CaOx- and GM-CSG-induced macrophages were negative for M2 macrophage markers pSTAT6 and CD206. M-CSF macrophages were positive for both pSTAT6 and CD206. Cells were fixed and stained with rabbit anti-pSTAT6 (green) and mouse anti-CD206 (red) (40×, bar, 100 µm). Nuclei were counterstained by 4,6-diamidino-2-phenylindole (DAPI, blue). *N* = 3.

## Abbreviations

CaOx, calcium oxalate; CCL2 (MCP-1), chemokine (C-C motif) ligand 2 (monocyte chemoattractant protein-1); CCL22 (MDC), chemokine (C-C motif) ligand 22 (macrophage-derived chemokine); IL-1b, interleukin 1 beta; IL-1Ra, interleukin 1 receptor antagonist; IL-6, interleukin 6; IL-8, interleukin 8; IL-10, interleukin 10; IFNα2a, interferon alpha 2a; IFNβ, interferon beta; IFNγ, interferon gamma; K_2_Ox, potassium oxalate; LPS, lipopolysaccharides; qRT-PCR, quantitative real-time PCR; TLR-4, toll-like receptor 4; TNFa, tumor necrosis factor-alpha; ZnOx, zinc oxalate.
