# Supplementary figures and images for: Calcium Oxalate Differentiates Human Monocytes Into Inflammatory M1 Macrophages
Source: Front Immunol. 2018 Aug 22;9:1863. doi: 10.3389/fimmu.2018.01863 (PMC6113402; doi:10.3389/fimmu.2018.01863)

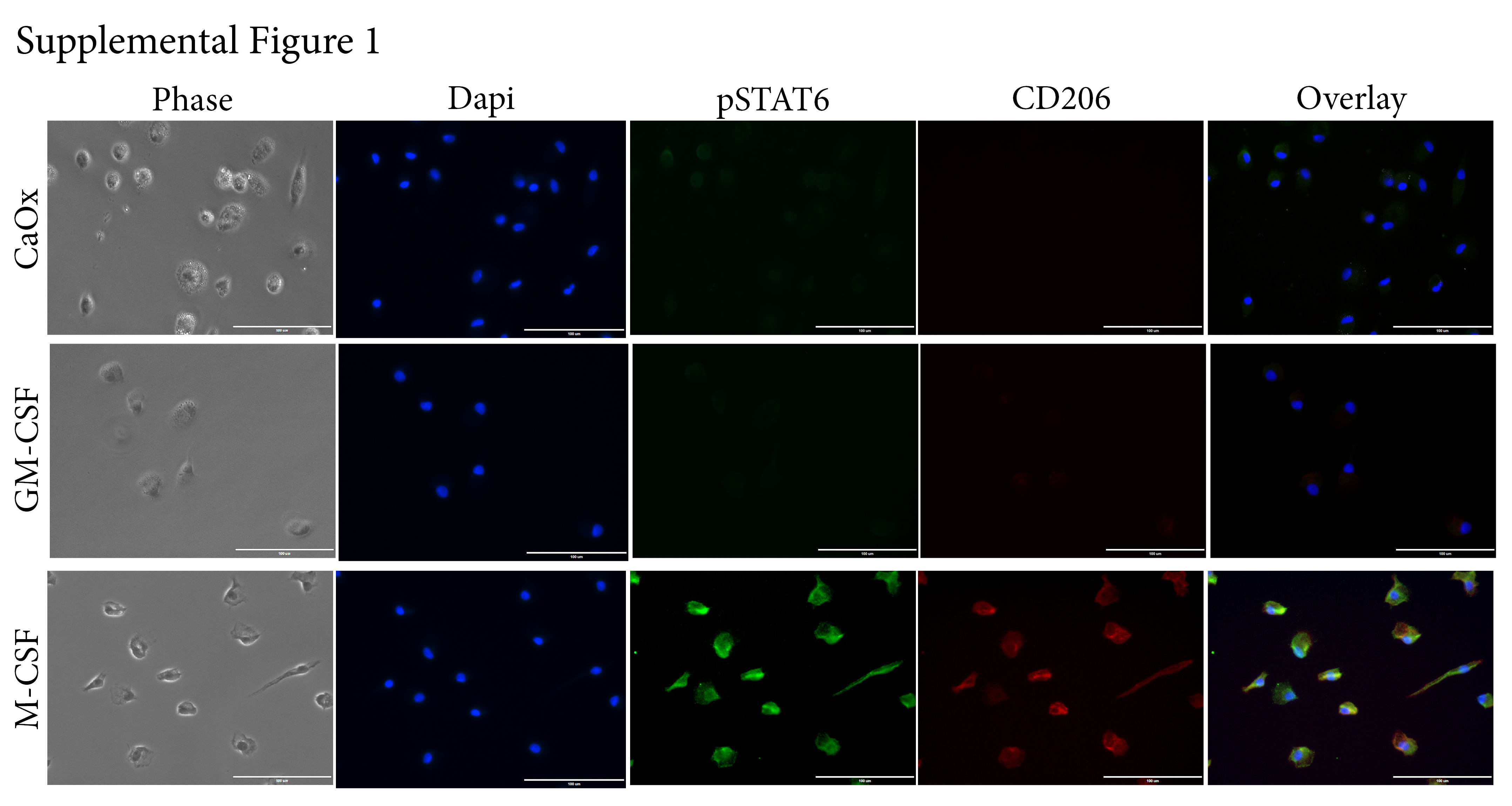

Supplement: Figure S1 — Calcium oxalate (CaOx) induced macrophages lack M2 macrophage markers phosphorylated STAT6 and CD206. CaOx- and GM-CSG-induced macrophages were negative for M2 macrophage markers pSTAT6 and CD206. M-CSF macrophages were positive for both pSTAT6 and CD206. Cells were fixed and stained with rabbit anti-pSTAT6 (green) and mouse anti-CD206 (red) (40×, bar, 100 µm). Nuclei were counterstained by 4,6-diamidino-2-phenylindole (DAPI, blue). N = 3. [file image_1.tiff]
